# XGBoost-SHAP-based interpretable diagnostic framework for alzheimer’s disease

**DOI:** 10.1186/s12911-023-02238-9

**Published:** 2023-07-25

**Authors:** Fuliang Yi, Hui Yang, Durong Chen, Yao Qin, Hongjuan Han, Jing Cui, Wenlin Bai, Yifei Ma, Rong Zhang, Hongmei Yu

**Affiliations:** 1 Department of Health Statistics, School of Public Health, Shanxi Medical University, 56 South XinJian Road, Taiyuan, 030001 P.R. China; 2 Shanxi Provincial Key Laboratory of Major Diseases Risk Assessment, Taiyuan, China

**Keywords:** Alzheimer’s disease, Machine learning, Imbalanced classes, Multiclassification, Interpretable framework, XGBoost-SHAP

## Abstract

**Background:**

Due to the class imbalance issue faced when Alzheimer’s disease (AD) develops from normal cognition (NC) to mild cognitive impairment (MCI), present clinical practice is met with challenges regarding the auxiliary diagnosis of AD using machine learning (ML). This leads to low diagnosis performance. We aimed to construct an interpretable framework, extreme gradient boosting-Shapley additive explanations (XGBoost-SHAP), to handle the imbalance among different AD progression statuses at the algorithmic level. We also sought to achieve multiclassification of NC, MCI, and AD.

**Methods:**

We obtained patient data from the Alzheimer’s Disease Neuroimaging Initiative (ADNI) database, including clinical information, neuropsychological test results, neuroimaging-derived biomarkers, and APOE-ε4 gene statuses. First, three feature selection algorithms were applied, and they were then included in the XGBoost algorithm. Due to the imbalance among the three classes, we changed the sample weight distribution to achieve multiclassification of NC, MCI, and AD. Then, the SHAP method was linked to XGBoost to form an interpretable framework. This framework utilized attribution ideas that quantified the impacts of model predictions into numerical values and analysed them based on their directions and sizes. Subsequently, the top 10 features (optimal subset) were used to simplify the clinical decision-making process, and their performance was compared with that of a random forest (RF), Bagging, AdaBoost, and a naive Bayes (NB) classifier. Finally, the National Alzheimer’s Coordinating Center (NACC) dataset was employed to assess the impact path consistency of the features within the optimal subset.

**Results:**

Compared to the RF, Bagging, AdaBoost, NB and XGBoost (unweighted), the interpretable framework had higher classification performance with accuracy improvements of 0.74%, 0.74%, 1.46%, 13.18%, and 0.83%, respectively. The framework achieved high sensitivity (81.21%/74.85%), specificity (92.18%/89.86%), accuracy (87.57%/80.52%), area under the receiver operating characteristic curve (AUC) (0.91/0.88), positive clinical utility index (0.71/0.56), and negative clinical utility index (0.75/0.68) on the ADNI and NACC datasets, respectively. In the ADNI dataset, the top 10 features were found to have varying associations with the risk of AD onset based on their SHAP values. Specifically, the higher SHAP values of *CDRSB*, *ADAS13*, *ADAS11*, *ventricle volume*, *ADASQ4*, and *FAQ* were associated with higher risks of AD onset. Conversely, the higher SHAP values of *LDELTOTAL*, *mPACCdigit*, *RAVLT_immediate*, and *MMSE* were associated with lower risks of AD onset. Similar results were found for the NACC dataset.

**Conclusions:**

The proposed interpretable framework contributes to achieving excellent performance in imbalanced AD multiclassification tasks and provides scientific guidance (optimal subset) for clinical decision-making, thereby facilitating disease management and offering new research ideas for optimizing AD prevention and treatment programs.

**Supplementary Information:**

The online version contains supplementary material available at 10.1186/s12911-023-02238-9.

## Background

Alzheimer’s disease (AD) is a progressive neurodegenerative disease that affects an estimated one out of nine people over 65 years of age worldwide [1–3]. The main clinical symptoms of AD include memory impairment, cognitive decline, and in severe cases, personality changes, loss of self-care ability, and possible fatality [4]. The prodromal status of AD is mild cognitive impairment (MCI), which is more pronounced than normal age-related decline but with preserved functional abilities [5], and this condition requires early intervention. According to the World Alzheimer Report 2021, the number of patients with AD will reach 131 million globally, and the estimated cost will stand at one trillion dollars by 2050, placing a heavy financial burden on society [6]. However, no effective medications are currently available, and diagnosing people living with AD in a timely manner is paramount.

When making AD diagnoses, experienced neurologists normally consider examinations consisting of patient histories, neuropsychological tests, neuroimaging results, and gene markers [7]. The *Trail Making Test* (*TMT-B*), *Mini-Mental State Examination* (*MMSE*), *Alzheimer’s Disease Assessment Scale-Cognitive Behaviour* (*ADAS-cog*), *Rey Auditory Verbal Learning Test* (*RAVLT*), and *Functional Assessment Questionnaire* (*FAQ*) are common neuropsychological tests that help differentiate between various degrees of cognitive impairment [8]. Chandra et al. used magnetic resonance imaging (MRI) to illustrate distinct brain damage patterns that can differentiate AD from other brain disorders, brain abnormalities that are linked to an increased risk of progressing to AD from MCI, and other behavioural outcomes [9]. The apolipoprotein E ε4 (APOE-ε4) gene is an important risk factor for AD [10]. However, manual diagnostic approaches have been shown to be unsatisfactory [11]. Therefore, an improved diagnostic approach is needed.

Machine learning (ML) is used for data mining and classification, enabling generalization and optimization [12]. In most cases, supervised learning models, such as random forest (RF) and AdaBoost, and unsupervised learning models, such as recurrent neural networks (RNNs), are utilized to understand disease patterns and prognoses and have potential to perform clinical auxiliary diagnoses [13–15]. However, they are accompanied by some inherent limitations: (1) imbalanced outcome classes are responsible for skewed performance, translating to lower sensitivity and higher misdiagnosis rates; and (2) complex ML algorithms contribute to higher accuracy but also possess greater learning difficulties and more uninterpretable internal mechanisms. Two main strategies are available for dealing with imbalanced scenarios [16]. The first is a data-level approach, in which the given data are preprocessed into a balanced dataset for classification. The second is an algorithmic-level approach, where classifiers are adapted to handle imbalanced data; one such algorithm is extreme gradient boosting (XGBoost), which was proposed by Chen et al. This algorithm can better perform imbalanced multiclassification by weighting the minority class by changing the sample weight distribution [17]. Understanding how a model makes an accurate prediction plays a role in the interpretation of many applications [18]. Lundberg et al. introduced Shapley additive explanations (SHAP), a post hoc interpretable algorithm that uses additive attribution to convert SHAP values from the machine learning feature space to the clinical variable space. This transformation improves the interpretability of previously difficult-to-explain algorithms [19].

It would be of great significance if, by using ML in auxiliary diagnosis cases, skewed classification performance and clinical misdiagnoses could be avoided and clinical confidence in decision-making could be enhanced while the medical burden is eased. To this end, our study sought to develop an interpretable ML framework by connecting XGBoost to SHAP with various features (e.g., clinical information, neuropsychological tests, and neuroimaging-extracted biomarkers and gene markers). We believe that it will facilitate the multiclassification of imbalanced classes (normal cognition [NC], MCI and AD), output valuable classification features, and determine the directions and sizes of interpretable effects. In addition, we compared its performance with other algorithms, such as a RF, Bagging, AdaBoost, and a naive Bayes (NB) classifier, and an external dataset was used to further evaluate the important features that assist with multiclassification diagnoses concerning AD progression.

### Related works

Prior research on predicting AD risk factors utilized standard ML models. Lin et al. developed a method for calculating scores based on different modalities (MRI, positron emission tomography [PET], cerebral spinal fluid [CSF], and genes) and applied these scores as inputs to an extreme learning machine (ELM)-based decision tree classifier to distinguish between subjects with progressive and stable MCI [20]. The suggested method was validated using the Alzheimer’s Disease Neuroimaging Initiative (ADNI) cohort, achieving an accuracy of 84.7% in terms of predicting AD within a timeframe of 3 years. Tufail et al. proposed a 2D classification architecture that utilizes multiple separable convolutional layers to differentiate between healthy individuals and patients with AD by analysing cross-sectional structural MRI (sMRI) images [21]. The architecture they constructed using the idea of cross-validation exhibited a fluctuating accuracy between 0.62 and 0.65. However, these two studies involved binary classification; they failed to realize the multiclassification task for AD, which remains a challenge.

Akter et al. explored state-of-the-art resampling techniques, including random oversampling, random undersampling, synthetic minority oversampling technique-adaptive synthetic sampling (SMOTE-ADASYN), SMOTE-Tomek, and SMOTE-edited nearest-neighbour (SMOTE-ENN) sampling, to handle severely imbalanced datasets before developing a novel hybrid ML model, AD-CovNet, which employs a long short-term memory-multilayer perceptron (LSTM-MLP) approach to identify AD in patients with and without COVID-19 [22]. In addition, Lin et al. proposed a linear discriminant analysis (LDA) method fusing multimodal data such as PET, MRI, CSF, and gene data and utilized an ELM-based decision tree approach for the multiclassification of NC, MCI, and AD by fusing data as new predictions [23]. However, the categories in this study were imbalanced (200 individuals with NC, 441 with MCI, and 105 with AD), no resampling techniques were employed, and the performance of the ELM was not satisfactory, with accuracy and F1 score values of 66.7% and 0.649, respectively. Notably, both of these decision approaches based on resampling and non-resampling pose potential drawbacks for the multiclassification of AD progression, with the former potentially causing data leakage (overoptimism or overfitting) and the latter potentially reducing classification performance due to imbalanced classes. In recent years, with advancements in neuroimaging techniques leading to the availability of large-scale multimodal neuroimaging data, deep learning has become the leading focus of research on the early detection and automated classification of AD [24]. One prominent type of approach is a convolutional neural network (CNN), which has demonstrated excellent performance in terms of making decisions based on images. Instead of utilizing inputs based on vectors, a CNN captures the structural information among adjacent pixels and leverages the spatial information of images to extract features. This is achieved by organizing convolutional layers to construct a feature hierarchy for decision-making [25]. Basheera et al. utilized a CNN for the binary and multiclassification of NC, MCI, and AD based on segmented grey matter derived from MRI [26]. The study included a total of 4463 participants, and the accuracy rates for identifying different conditions were as follows: 100% for AD-NC, 96.2% for AD-MCI, 98.0% for NC-MCI, and 86.7% for AD-MCI-NC. Hu et al. used raw T1 images to train a CNN model to discriminate AD, frontotemporal dementia, and the corresponding normal controls, with an accuracy of 91.83% [27]. However, one of the problems brought along with the high accuracy of this CNN for deep learning concerns its inherent complexity, making it difficult to gain insights into the internal mechanisms and intuitively uninterpretable classification results.

In short, previous studies had the following limitations. First, they focused on binary classification and were unable to satisfy the need for the multiclassification of AD progression. Second, imbalanced datasets, if dealt with improperly, are responsible for overoptimism or overfitting. Third, while pursuing accuracy, these models generally ignore their own interpretability, causing a clinical decision-making crisis. A detailed summary of these studies is presented in Table 1.

### This work

The main contributions of this research can be summarized as follows.

To realize multiclassification for NC, MCI, and AD, we utilized XGBoost with 3-fold cross-validation using the multiclassification strategy (one vs. rest) to transform these ML models into a multiclassification architecture.

We exploited the ability of the XGBoost algorithm, that is, we changed the sample weight distribution by tuning its hyperparameter (enhancing the weight of the minority class) to achieve a lower misdiagnosis rate and more accurate classification without other resampling techniques, which may lead to data leakage.

We combined XGBoost and SHAP to construct an interpretable ML framework, which improved the interpretability of the model and detected important features affecting the diagnosis of AD. Moreover, we sought an external dataset to validate the consistency of the clinically meaningful subset output by the framework to improve clinicians’ confidence in the decision-making results.

**Table 1:** Summary of related works

Authors	Method	Subjects	Modalities	Performance		Limitation
Lin et al. (2020) [20]	ELM	110 pMCI vs. 205 sMCI	MRI, PET, CSF, and genes	ACC: 84.7%		The multiclassification task for AD failed.
Tufail et al. (2020) [21]	DL	90 nonAD vs. 90 AD	sMRI	Fluctuated accuracy: 0.62–0.65		
Akter et al. (2022) [22]	SMOTE-ENN + AD-CovNet (long short-term memory-multilayer perceptron)	754 AD individuals with and without COVID-19	Demographic and clinical from medical records	ACC: 86% AUC: 0.857		The method of imbalanced data potentially causes data leakage (overoptimism or overfitting).
Lin et al. (2021) [23]	LDA + ELM	200 NC vs. 441 MCI vs. 105 AD	MRI, PET, CSF, and genes	ACC: 66.7% F1 score: 0.649		The 3 way classification performance is poor due to imbalanced problem.
Basheera et al. (2020) [26]	CNN	28 NC vs. 32 MCI vs. 65 AD	MRI	ACC	AD-NC: 100% AD-MCI: 96.2% NC-MCI: 100% AD-MCI-NC: 86.7%	The internal mechanism is complex and poorly interpretable.
Hu et al. (2021) [27]	DL-based networks	823 NC vs. 552 FTD vs. 422AD	MRI	ACC	FTD vs. FTD_NC: 93.45% AD vs. AD_NC: 89.86% FTD vs. AD vs. NC: 91.83% FTD vs. AD: 93.05%	

ELM: extreme learning machine; DL: deep learning; SMOTE-ENN: synthetic minority oversampling technique-edited nearest neighbour; LDA: linear discriminant analysis; CNN: convolutional neural network; FTD: Frontotemporal dementia; pMCI: progressive MCI; sMCI: stable MCI; ACC: accuracy; AUC: area under the receiver operating characteristic curve

## Methods

### Data sources

The data used in this study were obtained from the Alzheimer’s Disease Neuroimaging Initiative (ADNI) database (adni.loni.usc.edu) and the National Alzheimer’s Coordinating Center (NACC) (https://naccdata.org/).

The ADNI was launched in 2003 as a public-private partnership led by principal investigator Weiner. The primary goal of the ADNI is to test whether serial MRI, PET, other biological markers, and clinical and neuropsychological tests can be combined to measure the progression of MCI and early AD. The NACC, established in 1999 by the National Institute on Aging/NIH, aims to facilitate collaborative research. It has developed and maintained a large relational database containing standardized clinical and neuropathological research datasets.

### Data preprocessing and feature selection

The data acquired from the ADNI with a deadline of November 2021 contained subjects from ADNI 1, ADNI GO, ADNI 2, and ADNI 3. As a baseline, 714, 247, and 379 individuals were diagnosed with AD, MCI, and NC, respectively. We included 42 features, including patients’ clinical information, neuropsychological tests, neuroimaging-extracted biomarkers, and the APOE-ε4 gene. To ensure the integrity of the data, we removed features with missing value rates that were greater than 50% (14 features were excluded), and the remaining individuals with all features were included. Then, we applied information gain, Boruta, and elastic net to conduct feature selection for the most relevant features while eliminating redundant and correlated features. We selected the final features from those with the majority of votes from all three selection methods. Finally, we obtained data from 547 individuals, including 189 with NC, 302 with MCI, and 56 with AD; we also obtained 27 features, including four demographic features, 15 neuropsychological tests, seven neuroimaging-extracted biomarkers, and the APOE-ε4 gene.

NACC data from 349 individuals were used as the external set containing nine features, which comprised three diagnoses: 171 patients with NC, 70 with MCI, and 108 with dementia due to AD. Notably, among the nine features we included, four were demographic features that were equivalent to the demographic features of the ADNI dataset, while the other five were obtained by matching the top 10 important features in the dataset. Table 2 provides comprehensive information about all features contained in the two datasets.

**Table 2:** Details of the features used on the ADNI and NACC datasets

Features	ADNI	NACC
Demographic information	*AGE* (Subject’s age)	*NACCAGE*
	*PTGENDER* (Subject’s sex)	*SEX*
	*PTEDUCAT* (Years of education)	*EDUC*
	*PTMARRY* (Marital status)	*MARISTAT*
Gene	*APOE4* (Number of APOE-ε4 alleles)	
Neuropsychological tests	*ADAS11* (Alzheimer’s Disease Assessment Scale-Cognition 11 items)	
	*ADAS13* (Alzheimer’s Disease Assessment Scale-Cognition 13 items)	
	*ADASQ4* (Score from Task 4 (Word Recognition) of the Alzheimer’s Disease Assessment Scale)	
	*MMSE* (Total Score of Mini-Mental State Examination)	*NACCMMSE*
	*FAQ* (Total Score of Functional Activities Questionnaire)	*FAQ-sum*
	*MOCA* (Total Score of Montreal Cognitive Assessment)	
	*CDRSB* (Clinical Dementia Rating-Sum of Boxes Score)	*CDRGLOB*
	*RAVLT_immediate* (Rey’s Auditory Verbal Learning Test_Immediate Recall)	
	*RAVLT_learning* (Rey’s Auditory Verbal Learning Test_Learning)	
	*RAVLT_forgetting* (Rey’s Auditory Verbal Learning Test_Forgetting)	
	*RAVLT_perc_forgetting* (Rey’s Auditory Verbal Learning Test_Percent Forgetting)	
	*LDELTOTAL* (Delayed total recall)	*MEMUNITS*
	*TRABSCOR* (Trail Making Test Part B Time)	
	*mPACCdigit* (Modified Preclinical Alzheimer Cognitive Composite with Digit test)	
	*mPACCtrailsB* (Modified Preclinical Alzheimer Cognitive Composite with Trails test)	
Neuroimaging-extracted biomarkers	*Ventricles* (Volume of ventricles)	*LATVENT + HIRVENT*
	*Hippocampus* (Volume of hippocampus)	
	*WholeBrain* (Volume of Whole Brain)	
	*Entorhinal* (Volume of entorhinal)	
	*Fusiform* (Volume of fusiform)	
	*MidTemp* (Volume of middle temporal gyrus)	
	*ICV* (Volume of intracranial)	

### Construction of the XGBoost-SHAP framework

XGBoost is an improved gradient boosting algorithm that incorporates a regression tree. The idea of XGBoost is to iteratively add trees by learning the negative gradient of the loss function between the value predicted by the previous tree and the true value, and feature splitting is also continuously performed to grow an ensemble tree [28, 29]. This algorithm learns the negative gradient using the second-order derivative of the loss function, which enables faster convergence to global optimality and improves efficiency [30]. Furthermore, the algorithm introduces a penalty term for regularization to prevent overfitting. It can also maintain a balance between the negative and positive weights of classes by calculating the inverse of the ratio of negative to positive samples as a weighting operator, thus outshining many other algorithms.

The understandability of a prediction model is important in clinical practice. The interpretability of a model provides insights into the internal mechanisms of how it works. As a model-agnostic explanation approach, SHAP aids in interpreting predictive models such as XGBoost [31]. It assumes that each feature represents a “contributor” to the predictions of the XGBoost model and assigns them SHAP values; that is, the final prediction can be interpreted as the sum of the SHAP values of all features and the average prediction. SHAP transforms XGBoost’s feature space into a clinical variable space, where each transformed SHAP value corresponds to an original variable. SHAP usually graphically visualizes XGBoost predictions for a better presentation effect. For example, the SHAP summary plot offers a concise demonstration of the magnitudes and directions of predictions. The size of a SHAP value represents the contribution of one specific feature towards prediction performance: the larger the value, the higher the contribution [32]. The SHAP dependency plot depicts the SHAP value distribution across individuals for a feature. As the SHAP values of features vary between individuals, so do the predictions of the corresponding feature mappings for individuals.

In this study, we first tuned the hyperparameter range, after which a grid search was used for the best values to maximize the performance of XGBoost, thereby achieving multiclassification of the NC, MCI, and AD patients in the ADNI and NACC datasets. As the weights of the classes differed between the two datasets, we manually tuned the *scale_pos_weight* parameter. The ADNI dataset contained 189, 302, and 56 NC, MCI, and AD samples, respectively. The weight of the NC samples was set to 1, and the weight of the MCI samples was calculated to be approximately 0.62 (189/302), while the weight of the AD samples was approximately 3.4 (189/56). Similarly, the NACC dataset included 171, 70, and 108 NC, MCI, and AD samples, respectively. The weight of the NC samples was set to 1, and the weight of MCI samples was calculated to be approximately 2.5 (171/70), while the weight of AD samples was approximately 2 (171/108). Detailed information can be found in Table 3.

**Fig. 1:**
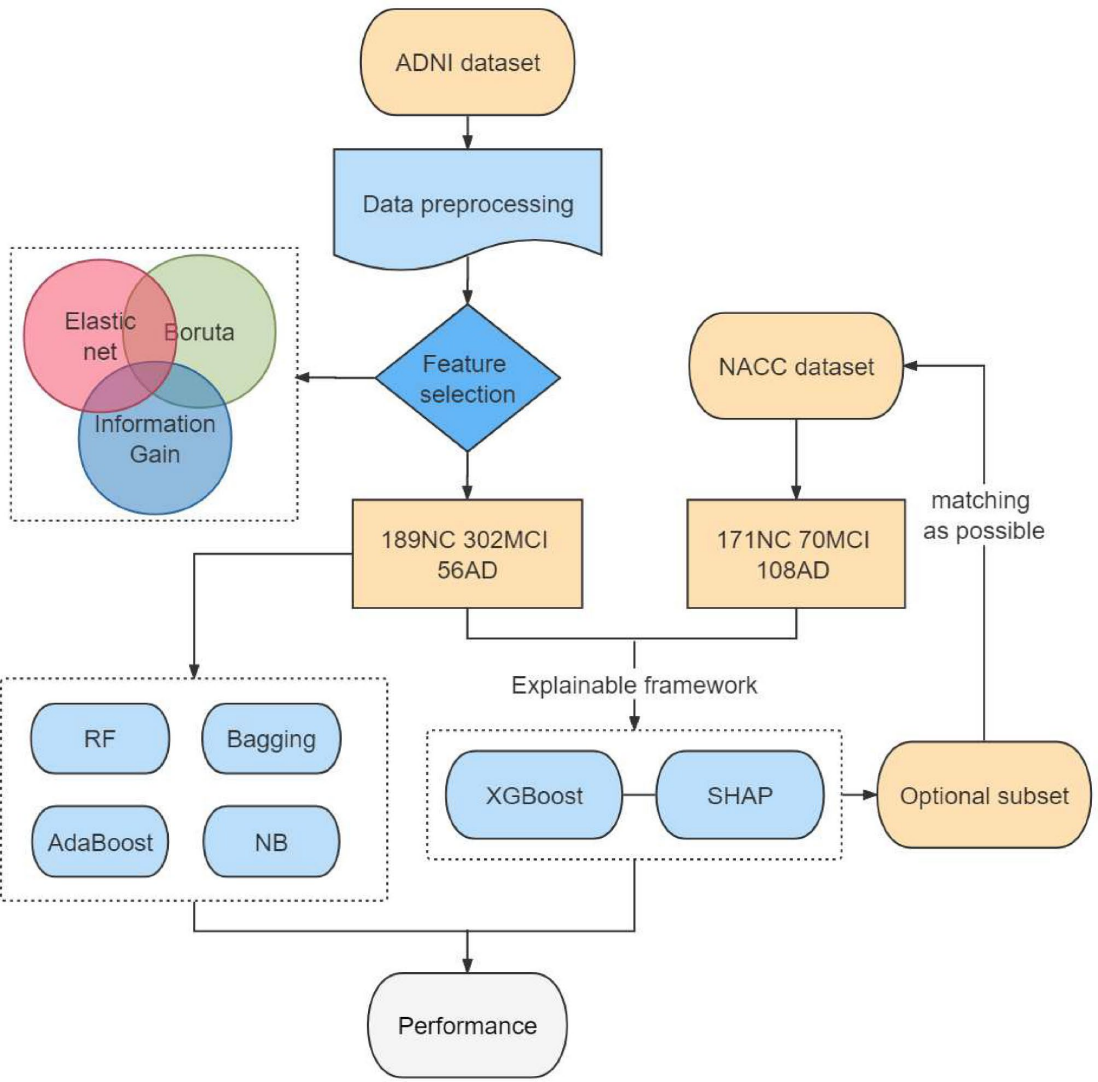
The flow chart of this study

Second, we linked SHAP to XGBoost to form an interpretable framework and printed the top 10 features based on their SHAP values. The output of the framework was visualized using the SHAP summary and dependency plots. The XGBoost-SHAP framework’s performance was compared to that of a RF, Bagging, AdaBoost, and a NB classifier. The details of these comparison algorithms are provided in the supplementary materials 1. Figure 1 presents a flowchart of the study.

The analyses were performed using R 4.1.1. Feature selection was performed using the following packages: “glmnet,” “Boruta,” and “FSelector.” The classification algorithms were implemented using “xgboost,” “randomForest,” “adabag,” and “e1071.” The SHAP analysis was carried out using the “SHAPforxgboost” package.

**Table 3:** Hyperparameters tuning of XGBoost

Range of hyperparameters	Hyperparameters on ADNI dataset	Hyperparameters on NACC dataset
*Booster = gbtree*	*Booster = gbtree*	*Booster = gbtree*
*eta* = (0.01: 0.3)	*eta* = 0.01	*eta* = 0.01
*gamma* = (0.5: 1)	*gamma* = 0.5	*gamma* = 0.5
*max_depth* = (5: 8)	*max_depth* = 5	*max_depth* = 5
*min_child_weight* = 1 (default)	*min_child_weight* = 1	*min_child_weight* = 1
*subsample* = 1 (default)	*subsample* = 1	*subsample* = 1
*colsample_bytree* = 1 (default)	*colsample_bytree* = 1	*colsample_bytree* = 1
*lambda =* (1–3)	*lambda =* 3	*lambda =* 3
*nrounds* = (75: 2000)	*nrounds* = 482	*nrounds* = 482
*scale_pos_weight*	*scale_pos_weight =* (1, 0.62, 3.4)	*scale_pos_weight =* (1, 2.5, 2)

### Evaluation of the classification algorithms

The metrics used to evaluate the classification performance of the tested algorithms included sensitivity, specificity, accuracy, and the area under the receiver operating characteristic curve (AUC). All metrics were acquired after weighting the possible classification results, where the arithmetic mean values of each statistical index for the three categories were considered the final evaluation metrics. This was repeated for each of the three classes in both datasets. We also included the clinical utility index (CUI) to clinically evaluate the interpretable framework. The CUI is divided into two parts, positive (CUI+) and negative (CUI-), which are calculated as positive predictive value * (sensitivity/100) and negative predictive value * (sensitivity/100), respectively. The following recommended diagnostic interpretations consist of “excellent utility” (CUI ≥ 81%), “good utility” (CUI ≥ 64%), “satisfactory utility” (CUI ≥ 49%), and “poor utility” (CUI < 49%) [3].

## Results

### Demographics of the ADNI and NACC datasets

The statistical descriptions of the demographic information concerning both datasets are shown in Tables 4 and 5, including age, gender, educational years, and marital statuses. The age, educational years (*PTEDUCAT*), and marital statuses (*PTMARRY*) in the ADNI dataset statistically differed between groups, whereas in the NACC dataset, the differences between groups were only statistically significant for age (*NACCAGE*).

**Table 4:** Demographic information among individuals in ADNI dataset

Variable	NC (n = 189)	MCI (n = 302)	AD (n = 56)	*P*
*AGE* ^*^	70.3 (66.9, 75.0)	70.6 (64.8, 75.8)	73.2 (68.3, 79.3)	0.026
*PTGENDER* ^$^				0.054
Male	83 (43.9%)	166 (55.0%)	30 (53.6%)	
Female	106 (56.1%)	136 (45.0%)	26 (46.4%)	
*PTEDUCAT* ^*^	18.0 (16.0, 18.0)	16.0 (14.0, 18.0)	16.0 (14.0, 18.0)	0.005
*PTMARRY* ^$^				0.035
Other	138 (73.0%)	236 (78.1%)	50 (89.3%)	
Married	51 (27.0%)	66 (21.9%)	6 (10.7%)	

^*^ Median (P25, P75) expressed continuous variables, analysed using the Kruskal-Wallis *H* test. ^$^ Categorical variables were expressed as percentage (%) and the χ^2^ test was employed

**Table 5:** Demographic information among individuals in NACC dataset

Variable	NC (n = 171)	MCI (n = 70)	AD (n = 108)	*P*
*NACCAGE* ^*^	77.0 (69.0, 85.0)	82.0 (76.2, 87.0)	81.0 (77.8, 86.0)	< 0.001
*SEX* ^$^				0.079
Male	55 (32.2%)	28 (40.0%)	49 (45.4%)	
Female	116 (67.8%)	42 (60.0%)	59 (54.6%)	
*EDUC* ^*^	15.0 (12.0, 18.0)	14.0 (12.0, 16.0)	14.0 (12.0, 17.0)	0.258
*MARISTAT* ^$^				0.340
Other	78 (45.6%)	34 (48.6%)	59 (54.6%)	
Married	93 (54.4%)	36 (51.4%)	49 (45.4%)	

^*^ Median (P25, P75) expressed continuous variables, analysed using the Kruskal-Wallis *H* test. ^$^ Categorical variables were expressed as percentage (%) and the χ^2^ test was employed

### Performance of the proposed models

**Fig. 2:**
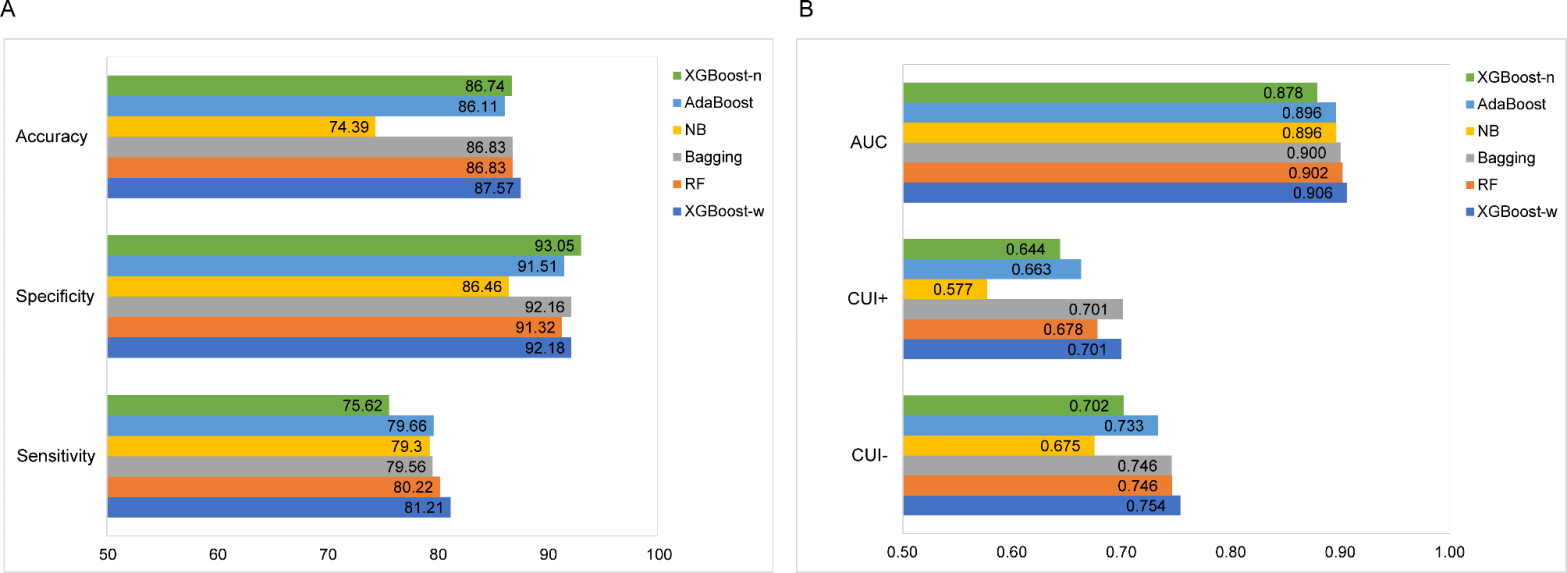
Performance metrics of XGBoost-n (unweighted), XGBoost-w (weighted), RF, Bagging, AdaBoost, and NB with 3-fold cross-validation strategy on the ADNI dataset

Figure 2-A-B shows the sensitivity, specificity, accuracy, AUC, CUI+, and CUI- of XGBoost and the other algorithms with respect to the multiclassification of NC, MCI, and AD. After conducting the comparison, we found that (1) XGBoost did not perform well before changing the sample weight distribution, yielding the lowest sensitivity and AUC; however, the sensitivity and AUC increased by 5.59% and 0.03, respectively, after changing the sample weight distribution, and the specificity decreased by only 0.87%. (2) XGBoost outperformed the other four algorithms in terms of classifying NC, MCI, and AD after adjusting the sample weights, exhibiting superior sensitivity, specificity, accuracy, and AUC. (3) Bagging and RF performed comparably, whereas AdaBoost and NB remained in need of improvement with sensitivity values lower than 80% and AUCs below 0.9. (4) Except for NB, all models offered good clinical utility (CUI ≥ 64%), with XGBoost being the best after modelling the sample weight distribution.

### Interpretable framework and feature importance

**Fig. 3:**
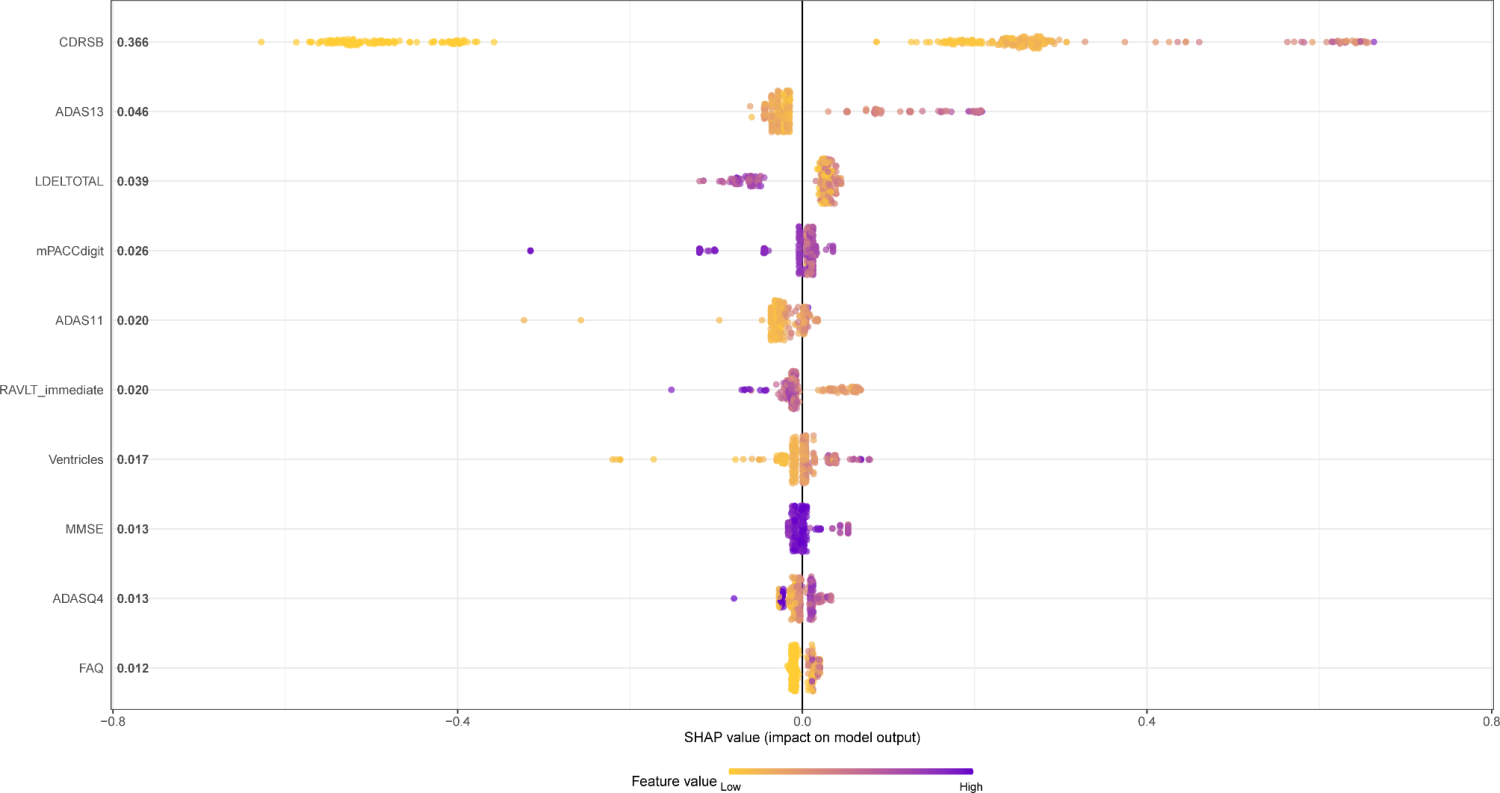
The SHAP summary plot for the top 10 important features in ADNI dataset

The SHAP summary plot in Fig. 3 shows the top 10 features produced by XGBoost in descending order according to the SHAP values of all predictions, which express the positive/negative associations of the corresponding features, with the absolute SHAP value for each feature shown on the left. Each point on the plot corresponds to a sample, and the horizontal axis indicates the SHAP value of a given feature across subjects, which reflects the magnitude of the SHAP value from low (yellow) to high (purple). Evidently, higher values of *CDRSB*, *ADAS13*, *ADAS11*, *volume of ventricles*, *ADASQ4*, and *FAQ* were associated with higher risks of AD onset, so they can be interpreted as risk factors for AD, while higher values of *LDELTOTAL*, *mPACCdigit*, *RAVLT_immediate*, and *MMSE* were affiliated with lower risks of AD onset, so they can be interpreted as protective factors.

**Fig. 4:**
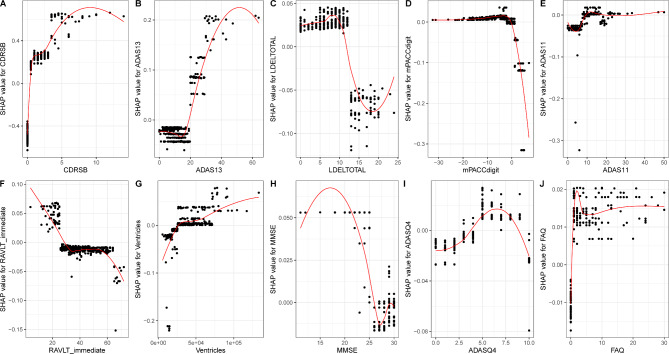
The SHAP dependence plots for the top 10 important features in ADNI dataset

With the SHAP value as the vertical axis and the feature value as the horizontal axis, SHAP dependence plots make the values of many individuals available in one plot, facilitating an up-and-down trend of feature-attributed importance. Figure 4 shows the SHAP dependence plots for the top 10 features. The value on the horizontal-axis represents the original value of a feature, whereas the value on the vertical-axis represents the SHAP value of a feature across individuals. Those with *CDRSB* scores of 10 exhibit higher SHAP values than those with scores of 1, indicating a higher likelihood of AD prediction. In contrast with *CDRSB*, a higher *LDELTOTAL* score equates to a lower SHAP value, and associated individuals are less likely to be diagnosed with AD.

In fact, SHAP summary and dependency plots are complementary in that the former can visually reflect the direction and magnitude of the effect of a feature contribution, whereas the latter can more clearly reflect the fluctuations exhibited by the SHAP values of a feature across individuals.

### Framework performance on the external dataset

After determining the performance of the interpretable XGBoost-SHAP framework on the ADNI dataset and conducting comparisons with other algorithms, we found that the framework works well for the multiclassification of NC, MCI, and AD. Therefore, we further evaluated the framework using an external dataset, particularly in terms of the effect of the optimal subset obtained from the ADNI dataset. As there were differences between the measures obtained on the two datasets, we matched the first 10 features that SHAP could offer, and features that failed to be matched were treated as missing variables. We matched five features: *CDRSUM* (matched to *CDRSB*), *NACCMMSE* (matched to *MMSE*), *MEMUNITS* (matched to *LDELTOTAL*), *FAQ-sum* (matched to *FAQ*), and *LATVENT + HIRVENT* (matched to the *volume of ventricles*).

**Fig. 5:**
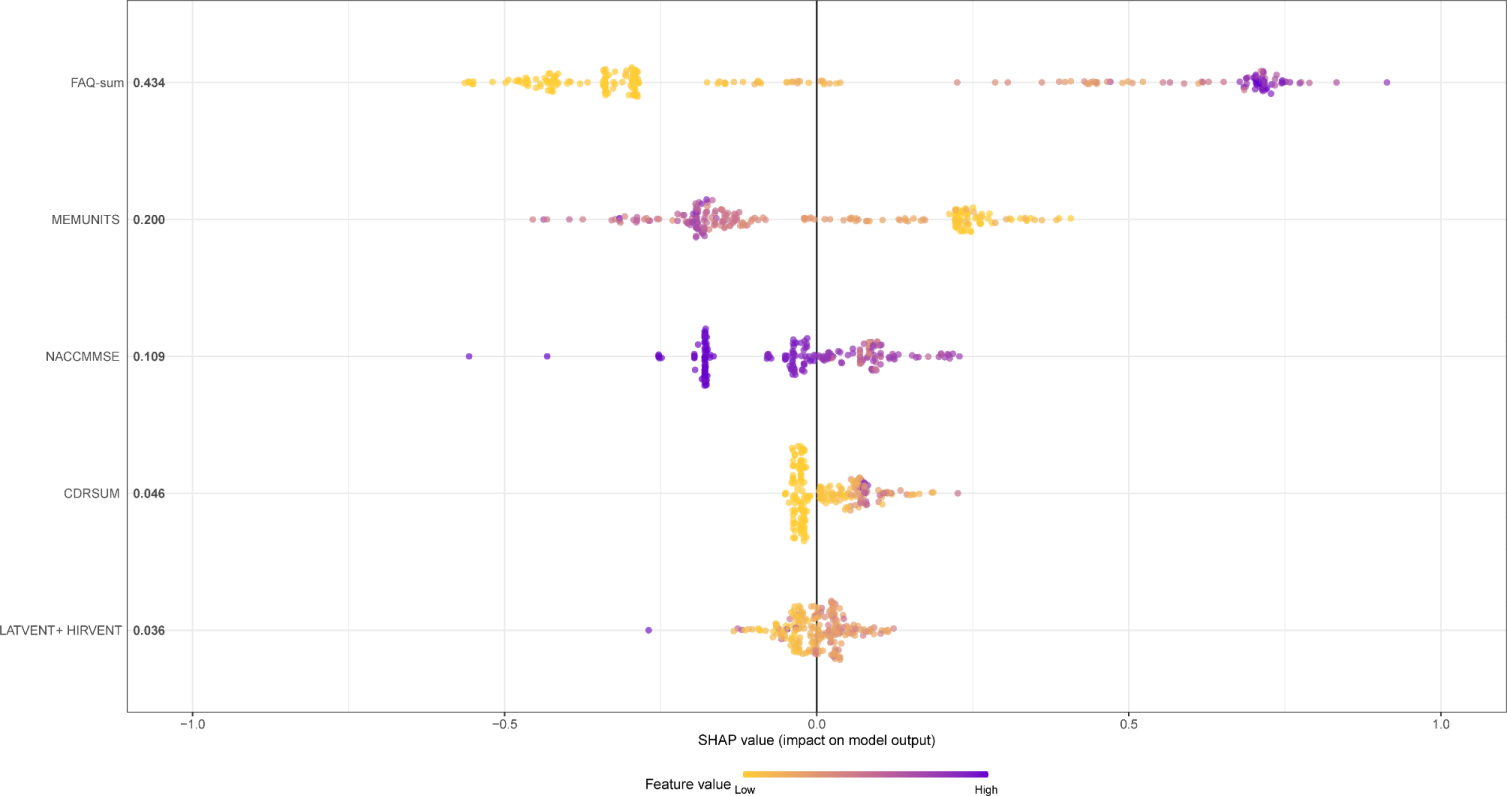
The SHAP summary plot for the five features matched in NACC dataset

**Fig. 6:**
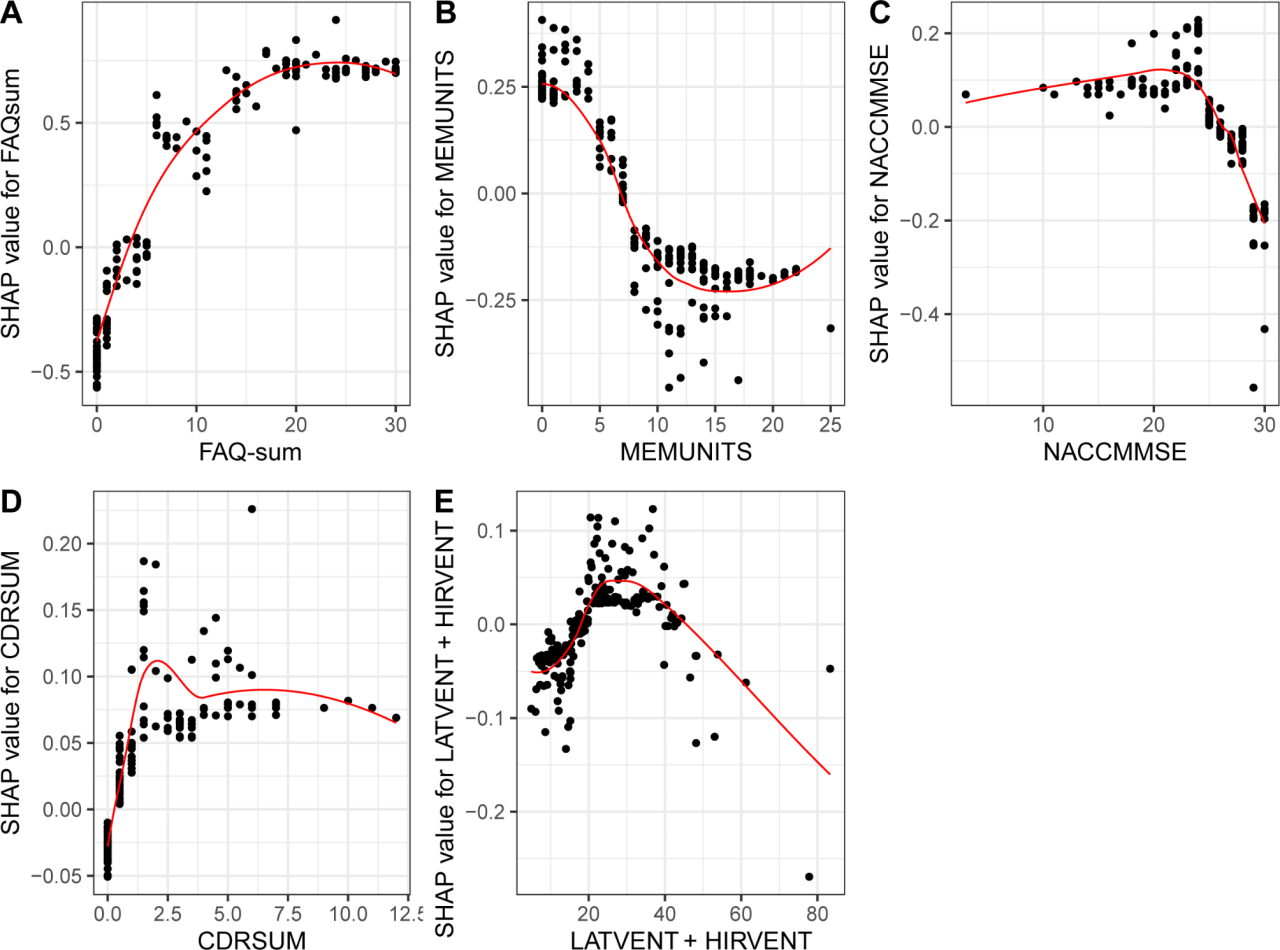
The SHAP dependence plots for the five features matched in NACC dataset

Subsequently, we fed these five features into the interpretable XGBoost-SHAP framework, yielding the following performance metrics. (1) The sensitivity, specificity, accuracy, AUC, CUI+, and CUI- of XGBoost reached 74.85%, 89.86%, 80.52%, 0.88, 0.56, and 0.68, respectively. (2) Similarly, we see in Figs. 5 and 6 that higher SHAP values of *FAQ-sum*, *CDRSUM*, and *LATVENT + HIRVENT* were associated with higher risks of AD onset, so these features can be interpreted as risk factors for AD, while higher values of *NACCMMSE* and *MEMUNITS* were associated with lower risks of AD onset, and these features can be interpreted as protective factors for AD. (3) We can conclude that individuals with *FAQ-sum* values of 20 have higher SHAP values than those with *FAQ-sum* values of 1; hence, the former individuals have higher AD prediction probabilities. In contrast to *FAQ-sum*, a higher *MEMUNITS* score equates to a lower SHAP value, and these individuals have lower AD prediction probabilities. It is noteworthy that the results are in line with both datasets used in this study.

## Discussion

This study developed an interpretable XGBoost-SHAP framework using the ADNI dataset, and the ability of this framework to perform multiclassification on three imbalanced classes (NC, MCI, and AD) was assessed by changing the distribution of the sample weights. We sought to determine the practical value of this framework as a clinical auxiliary diagnostic tool. External data from appropriately representative target-patient clinical cohorts are required to avoid overestimating the initial results, which leads to overfitting. Thus, we generalized this framework by matching the ADNI dataset as closely as possible with another dataset (NACC). Our results confirmed that the framework was stable for the two datasets and that consistent feature contribution directions were produced.

Several issues were identified during this process. As these two datasets involved different population studies and disease testing centres, they were subject to subtle differences in their diagnostic outcomes. Certain ADNI dataset features were not present in the external dataset. However, we looked at the *Researchers Data Dictionary* of the NACC-UDS to match the features as closely as possible. The following are some examples: *CDRSUM* matched with *CDRSB*, *NACCMMSE* matched with *MMSE*, *MEMUNITS* matched with *LDELTOTAL*, *FAQ-sum* matched with *FAQ*, and *LATVENT + HIRVENT* matched with the *volume of ventricles*. More importantly, we used the same parameters in both datasets to avoid between-dataset fluctuations among the features and classes as much as possible, as these could affect the usefulness of the algorithm. The parameter tuning process is described in detail in the **“Construction of the XGBoost-SHAP framework”** section.

ML is data-driven and may be beset by imbalanced outcomes. Prediction models established with imbalanced datasets are most frequently subjected to the majority class, meaning that there is a high risk of misclassifying minority examples; avoiding this bias is extremely crucial [33]. This also applies to AD diagnosis, especially with traditional ML models, because they operate under the assumption that the classification error costs are the same [34–36]. To address these concerns, researchers typically employ resampling techniques, including undersampling, oversampling, and mixed sampling, followed by cross-validation for algorithm training and performance evaluation purposes [37–40]. Vinutha et al. improved the AD diagnosis performance by handling imbalanced data and demonstrating the performance of the SMOTE [41]. Additionally, five methods for imbalanced data, the SMOTE, Borderline-SMOTE, support vector machine SMOTE (SVMSMOTE), ADASYN, and SMOTE-Tomek, were examined by Bogdanovic et al., who suggested that the SMOTE method was best. Subsequently, XGBoost was used to classify the participants into five categories (NC, SMC, early MCI, late MCI, and AD) with an accuracy of 0.84 [42]. In addition, Dubey et al. concluded that an ensemble system comprising sparse logistic regression with robustness selection as a feature selection algorithm and the K-medoids complete undersampling approach excellently addressed the class imbalance issue associated with the ADNI dataset. The results demonstrated that the accuracy of NC vs. MCI Converter & AD based on SVM majority voting amounted to 0.85 [43]. However, the above strategies were first used to resample the entire dataset to achieve a completely balanced class distribution; then, cross-validation was applied, which could easily lead to potential data leakage (overoptimism) [44].

This study exploited the inherent parameter tuning of XGBoost to enhance the weight of the minority class (AD) by setting the weights of NC, MCI, and AD to 1, 0.62, and 3.4, respectively. As such, we achieved multiclassification without any resampling techniques that may have led to overoptimism or overfitting. Additionally, our study achieved better results than XGBoost without changing the sample weight distribution, as represented by sensitivity, accuracy, and AUC increases of 5.59%, 0.83%, and 0.03, respectively. Our results were superior to those of similar previous studies.

As Tsoy et al. suggested, collecting and measuring numerous neuropsychological tests as well as neuroimaging examinations is extremely challenging, as they are resource-intensive, time-consuming, and expensive [45]. A constructive subset of features can greatly ease the work of clinicians by algorithmically identifying the variables that play decisive roles in the classification process to facilitate rapid clinical diagnosis while also ensuring that the patient’s fatigue and burden are minimized. In our study, we maintained accurate detection rates while identifying key measures (“features”) to improve the effectiveness of dementia diagnosis wherever possible. According to the SHAP values, *CDRSB*, *ADAS13*, and *LDELTOTAL* were considered the most important features, followed by *mPACCdigit*, *ADAS11*, *RAVLT_immediate*, *volume of ventricles*, *MMSE*, *ADASQ4*, and *FAQ*, in terms of identifying NC, MCI, and AD. In other words, the above features can be considered an optimal subset that represents the major players in the auxiliary diagnosis regarding the multiclassification of AD processes. The optimal subset is interpretable because the effect direction of each feature and the size of its contribution to the prediction are captured and visualized. Among these features, *CDRSB, ADAS13, ADAS11, volume of ventricles, ADASQ4, and FAQ* were positively associated with the occurrence of AD, whereas the others were negatively associated.

Next, we analysed the top three most significant features that were employed. *CDRSB*, *ADAS13* and *LDELTOTAL* constitute classic neuropsychological tests and are experiencing great popularity in clinical practice [46, 47]. *CDRSB* (ranging from 0 to 18) is a composite neuropsychological test that assesses both cognition (memory, orientation, judgement and problem solving) and function (community affairs, home and hobbies, personal care) and serves as a good candidate for predicting AD [48, 49]. *ADAS* was designed to evaluate both cognitive and noncognitive impairment in AD severity, where *ADAS-cog* is the more commonly utilized measure. *ADAS13* (ranging from 0 to 85) builds upon *ADAS11* by including two extra tasks, delayed recall and digital cancellation, thereby achieving improved sensitivity to early AD progression [50–52]. *LDELTOTAL* (ranging from 0 to 25) is a revised version of the episodic memory assessment found in the Wechsler Memory Scale-Revised, which measures a subject’s ability to recall a short story that contains 25 items of information after a delay of 30 to 40 min [53]. The results are reflectors of cognitive and social functioning, and delayed recall. This is important for identifying people with AD, as they typically experience cognitive decline, decreased daily living activities, and impaired episodic memory [54, 55]. Other neuropsychological tests in the optimal subset also identify disease progression in the domains of cognition, memory, and independent living. The neuroimaging-extracted biomarkers in our study, however, did not exhibit great potential for identifying NC, MCI, and AD. Only one feature, the *volume of ventricles*, was part of the optimal subset, but it did not contribute significantly (SHAP value of 0.017), which is in accordance with the results of Sanjay et al. [2]. One explanation for this finding relates to the unclear early changes in the brain anatomy in patients with MCI. Neuroimaging-extracted biomarkers can readily distinguish AD from NC but might not readily distinguish between NC and MCI [56].

### Limitations

Our study had several limitations. First, suitable variables were unavailable for matching *ADAS13*, *ADAS11*, *ADAQ4*, *mPACCdigit*, and *RAVLT_immediate* in the NACC dataset. Since only some of the participants completed *RAVLT_learning*, we deleted it, although it enjoys some diagnostic significance in clinical practice. Therefore, it was impossible to validate the impacts of these features on the multiclassification results. In addition, more clinical parameters (e.g., drug use) and imaging features (e.g., diffusion tensor imaging and resting-state functional MRI) were not included in this study. Our future research will involve the use of additional relevant modalities and features to improve the interpretability of our framework and validate its AD multiclassification capabilities on appropriate datasets.

## Conclusions

In real-world clinical studies, it is common to classify diseases based on multiple outcomes. In this case, an imbalance between multiple classes is frequently observed, and if the relationship between resampling and modelling is not effectively handled before conducting training, it generally makes the obtained results overly optimistic and causes them to lose their authenticity. When generalizing a model, the performance drops significantly. The algorithmic level, where classifiers are adapted to handle imbalanced data, can facilitate classification performance. It is also important to understand how an effective algorithm works. The interpretable framework that we constructed, XGBoost-SHAP, perfectly handled the above defects. It not only achieved AD multiclassification with imbalanced classes by changing the sample weight distribution but also explained the directions and sizes of the features and optimized the required features during the classification process; this will help clinicians make decisions. This framework offers a broad approach for connecting machine learning to disease pathophysiology in a generalizable manner. Based on the results of our study, we believe that the proposed interpretable framework, XGBoost-SHAP, can be effectively applied to imbalanced clinical and imaging data, making it a valuable clinical tool for the early detection of AD. Our ongoing work should emphasize the validation of the proposed interpretable framework by using more modalities and features that are important to AD multiclassification and exploring more AD-related features that matter to early AD screening.

## Electronic supplementary material

Below is the link to the electronic supplementary material.

Supplementary Material 1

## Data Availability

The data supporting the findings of this study are available from the Alzheimer’s Disease Neuroimaging Initiative (ADNI) database (adni.loni.usc.edu) and the National Alzheimer’s Coordinating Center (NACC) (https://naccdata.org/). To obtain access to the data, users must submit a request to the ADNI and NACC database managers.

## Abbreviations

AD: Alzheimer’s disease
NC: Normal cognition
MCI: Mild cognitive impairment
ML: Machine learning
AUC: Area under the receiver operating characteristic curve
CUI: Clinical utility index
SHAP: Shapley additive explanations
APOE-ε4: Apolipoprotein E ε4
XGBoost: Extreme gradient boosting
ADNI: Alzheimer’s Disease Neuroimaging Initiative
NACC: National Alzheimer’s Coordinating Center
