# Supplementary material for: XGBoost-SHAP-based interpretable diagnostic framework for alzheimer’s disease
Source: BMC Med Inform Decis Mak. 2023 Jul 25;23:137. doi: 10.1186/s12911-023-02238-9 (PMC10369804; doi:10.1186/s12911-023-02238-9)
Supplement: Supplementary file 1 — Supplementary Material 1 [file 12911_2023_2238_MOESM1_ESM.docx]

**Supplementary material for other algorithms we utilized**

**Bagging**, which is also known as bootstrap aggregation, is the ensemble learning method commonly used to reduce variance within a noisy dataset. In bagging, a random sample of dataset in a training set is selected with replacement-meaning that the individual dataset points can be chosen more than once. Once several dataset samples are generated, these weak models are then trained independently, and depending on the type of task-regression or classification, for example—the average or majority of those predictions yield a more accurate estimate [1]. Of note, the RF algorithm is considered an extension of the bagging method, using both bagging and feature randomness to create an uncorrelated forest of decision trees. A problem with decision trees like CART is that they are greedy. They choose which variable to split on using a greedy algorithm that minimizes error. As such, even with Bagging, the decision trees could have a lot of structural similarities and in turn, have a high correlation in their predictions. **Random forest (RF)** changes the algorithm for the way that the sub-trees are learned so that the resulting predictions from all of the subtrees have less correlation. Default tuning parameters were set in Bagging and random forest [2].

**AdaBoost**, also referred as Adaptive Boosting, is a Machine Learning technique that is utilized as an Ensemble Method. The most frequent AdaBoost algorithm is decision trees with one level, which is decision trees with only one split. These trees are often referred to as Decision Stumps. This algorithm constructs a model and assigns equal weights to all data points. It then applies higher weights to incorrectly categorized points. In the next model, all points with greater weights are given more weight. It will continue to train models until a smaller error is received [3].

**Naive Bayes (NB)** is a classification technique based on Bayes’ theorem with an assumption of independence among predictors. In simple terms, it assumes that the presence of a particular feature in a class is unrelated to the presence of any other feature and that is why it is known as ‘Naive’ [4]. NB model is easy to build and particularly useful for very large dataset sets. Along with simplicity, Naive Bayes is known to outperform even highly sophisticated classification methods. All parameters in NB model we constructed have defaulted.

Reference

1. Breiman L: Bagging predictors. *Machine learning* 1996, 24(2):123-140.

2. Gupta A, Kahali B: Machine learning‐based cognitive impairment classification with optimal combination of neuropsychological tests. *Alzheimer's & Dementia: Translational Research & Clinical Interventions* 2020, 6(1):e12049.

3. Keshvari S, Farizhendi SA, Ghiasi MM, Mohammadi AH: AdaBoost Metalearning Methodology for Modeling the Incipient Dissociation Conditions of Clathrate Hydrates. *ACS omega* 2021, 6(41):26919-26931.

4. Jiang W, Shen Y, Ding Y, Ye C, Zheng Y, Zhao P, Liu L, Tong Z, Zhou L, Sun S: A naive Bayes algorithm for tissue origin diagnosis (TOD‐Bayes) of synchronous multifocal tumors in the hepatobiliary and pancreatic system. *International journal of cancer* 2018, 142(2):357-368.
